# Microneedle-Mediated Transdermal Delivery of Biopharmaceuticals

**DOI:** 10.3390/pharmaceutics15010277

**Published:** 2023-01-13

**Authors:** Hiep X. Nguyen, Chien N. Nguyen

**Affiliations:** 1 Department of Pharmaceutical Sciences, College of Pharmacy, Mercer University, Atlanta, GA 30341, USA; 2 National Institute of Pharmaceutical Technology, Hanoi University of Pharmacy, Hanoi 100000, Vietnam; 3 Faculty of Pharmaceutics and Pharmaceutical Technology, Hanoi University of Pharmacy, Hanoi 100000, Vietnam

**Keywords:** microneedles, skin, biopharmaceuticals, drug delivery, stability

## Abstract

Transdermal delivery provides numerous benefits over conventional routes of administration. However, this strategy is generally limited to a few molecules with specific physicochemical properties (low molecular weight, high potency, and moderate lipophilicity) due to the barrier function of the stratum corneum layer. Researchers have developed several physical enhancement techniques to expand the applications of the transdermal field; among these, microneedle technology has recently emerged as a promising platform to deliver therapeutic agents of any size into and across the skin. Typically, hydrophilic biomolecules cannot penetrate the skin by passive diffusion. Microneedle insertion disrupts skin integrity and compromises its protective function, thus creating pathways (microchannels) for enhanced permeation of macromolecules. Microneedles not only improve stability but also enhance skin delivery of various biomolecules. Academic institutions and industrial companies have invested substantial resources in the development of microneedle systems for biopharmaceutical delivery. This review article summarizes the most recent research to provide a comprehensive discussion about microneedle-mediated delivery of macromolecules, covering various topics from the introduction of the skin, transdermal delivery, microneedles, and biopharmaceuticals (current status, conventional administration, and stability issues), to different microneedle types, clinical trials, safety and acceptability of microneedles, manufacturing and regulatory issues, and the future of microneedle technology.

## 1. Introduction

### 1.1. Skin Structure

**Figure 1:**
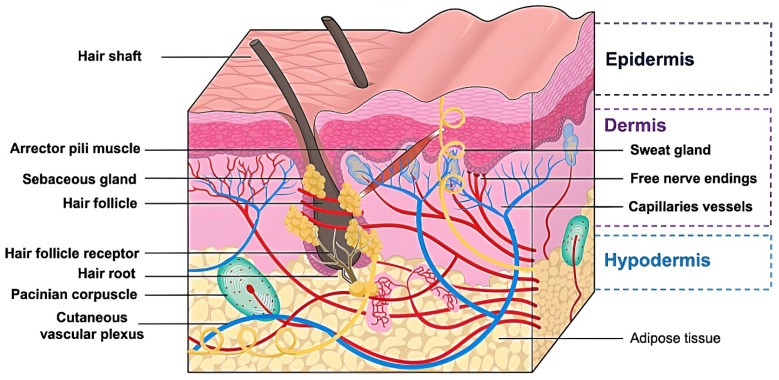
Schematic representation of human skin layers. Image reprinted with permission from [8].

The skin has been regarded as the body’s largest and most complicated organ, accounting for approximately 15% of the body mass and spanning an area of 1.5 to 2.0 sq.m. Evidently, the skin serves as the frontline protection and principal defense mechanism against detrimental environmental factors, including dehydration, the transmission of disease-causing microorganisms, physical, chemical, and biological stresses [1,2,3,4]. Furthermore, the skin also possesses several characteristics for safe and effective drug administration. Importantly, topical and transdermal delivery aims at the skin as the preferential absorption target of numerous pharmaceutical molecules. However, passive drug diffusion into the skin is generally impeded by the stratum corneum layer, which is the outermost lipophilic layer of the skin (20–50 µm thick) [5,6] (Figure 1). This tight layer contains dead keratinocytes (corneocytes), the intercellular lipid matrix, and corneodesmosome, thus forming the so-called ‘bricks and mortar’ structure in which the ‘bricks’ symbolize keratinized corneocytes and the ‘mortar’ embodies the continuous lipid matrix. Only moderately lipophilic compounds (log P of 1.0–3.0) could bypass the skin’s lipid-enriched structure to enter the underlying skin layers. The tight junction created by covalent bonds between the corneocytes and lipid matrix constitutes the primary protective barrier function of the skin, also known as the major rate-limiting barrier of drug delivery [7].

### 1.2. Transdermal Drug Delivery

The domains of pain management [9], hormone replacement treatment, central nervous system (CNS) disorders [10], hypertension, cardiovascular conditions, motion sickness, and smoking cessation strategies [11] have benefited greatly from the transdermal route of drug administration. In the upcoming years, the market for transdermal systems is expected to expand tremendously. Presently, there are only 20 compounds and 44 products (with varying concentrations) on the market that are approved by the US FDA for transdermal application [12]. In general, all transdermally administered pharmaceuticals exhibit three attributes: (i) low molecular weight, (ii) moderate lipophilicity, and (iii) high potency.

Typically, researchers reported drug permeation into and across the skin via three primary diffusion pathways, namely the transcellular, intercellular, and transappendageal pathways. For the transcellular pathway, permeants directly penetrate across the lipid bilayer membrane of the stratum corneum. This is the optimal delivery route for compounds with high lipophilicity. For the intercellular pathway, permeants travel along the tortuous and continuous intercellular lipid matrix surrounding the keratinocytes in the stratum corneum layer. Hydrophilic, uncharged, and low-molecular-weight compounds were found to enter the skin by this intercellular route [13]. Moreover, hair follicles and sweat glands, collectively known as the skin appendages (transappendageal pathway), are the preferential permeation pathways for several permeants, especially polar, ionizable, hydrophilic, and high-molecular-weight molecules. Several investigations have revealed the two main factors that determine how efficiently a substance is absorbed into the skin: (i) skin properties and (ii) physicochemical properties of the compound [14]. The rate and extent of drug permeation could be significantly affected by various skin factors such as thickness, composition, structure, age, species, application site and duration, disease conditions, hydration level, and skin treatment [15,16]. Regarding the permeants’ physicochemical properties, only a few selected molecules with specific features (molecular weight range of 100–500 Da, moderate lipophilicity with log P of 1.0–3.0) could enter the skin by passive diffusion [7]. A widely accepted principle indicates that passive drug diffusion is fueled and driven by the drug’s gradient concentration, thus being proportional to the drug levels in the applied formulation [17]. Additionally, the drug’s ionization degree has a substantial impact on the drug permeation efficiency. Moreover, a low melting point enables a significant enhancement in drug delivery into the skin. An ideal molecule for transdermal delivery should have a high potency with a low minimum effective dose. For instance, a required daily dose of 10 mg from a 10 sq.cm transdermal patch is desired [18].

As compared to traditional methods of drug administration, transdermal delivery provides several benefits. Patients, particularly young children and the elderly, are more likely to prefer transdermal products since the administration process is simple, noninvasive, and convenient [19,20,21]. Further advantages include the elimination of first-pass hepatic metabolism, the ability to provide sustained drug delivery and reduce administration frequency, the simplicity of application and termination, the convenience of access to the application site, the avoidance of requirements for healthcare professionals, the reduction in the required doses, the improvement in the drug’s bioavailability, and the prevention of any risk of disease transmission, thus offering a reliable alternative for those who do not favor conventional therapies [22,23,24]. In a recent review, Mohammed et al. presented different aspects of topical and transdermal delivery, including advantages, disadvantages, skin biology and conditions, and permeation enhancement strategies [25].

### 1.3. Microneedle Technology

An enhancement in transdermal and intradermal drug delivery could be achieved using a variety of strategies, including penetration enhancers, innovative formulation designs, and physical techniques [26]. Recently, microneedles have emerged as the most effective and reliable method for transdermal drug delivery, as recommended by multiple research works in academic institutions and industrial companies [4,27,28,29]. Micron-sized needles (Microneedles, 25–2000 µm long) have been reported to perforate skin layers to precisely and reversibly disrupt the skin barrier function, creating numerous microchannels in the skin [30].

Microneedle technology possesses a long history of more than 40 years of development. The concept of microscale needles first appeared in a patent authored by Gerstel and Place, and granted by the United States Patent and Trademark Office in 1976. The advancement in the microfabrication industry facilitated more precise and controlled fabrication of microneedles. The development of various microneedle types (i.e., solid, hollow, coated, dissolving, and swelling microneedles) followed, as presented chronologically below. In particular, the hydrogel-forming swelling microneedle, developed in 2012 by Donnelly and colleagues, is the latest design of microneedles for skin delivery. Recently, researchers have paid substantial attention to dissolving microneedles, inventing superior materials, developing novel designs, and optimizing scalable production techniques. A large volume of research led to the increased popularity of microneedles. This evolving field further expands to cover cosmetic and diagnostic applications, and drug delivery to various tissues (i.e., eye, buccal mucosa, gastrointestinal tract, etc.).

The first microneedle design was patented in 1976, followed by the patent of a hollow microneedle device for intradermal drug delivery in 1996. A skin-perforating device was developed in 1997, while silicon solid microneedles were first used for transdermal delivery of calcein in 1998. In 2000, researchers invented hollow microneedles to inject a drug solution into the skin. The first coated microneedles were fabricated in 2004 to enhance transdermal delivery of desmopressin. After that, in 2006, drug-loaded dissolving microneedles were fabricated to deliver bovine serum albumin and calcein transdermally. Lastly, hydrogel-forming swelling microneedles were invented in 2012 as the most recent microneedle type. 

Extensive reviews have been presented on various topics related to microneedles, including manufacturing processes [31,32], designs [33], applications in drug delivery, safety [34], clinical studies [31], modelling, simulation [35], and many more. Research findings have demonstrated that microneedles could puncture the skin without penetrating the dermis, which houses nerve fibers and blood vessels, to avoid causing pain or bleeding. Recently, Nguyen and colleagues reviewed the strategies of microneedle applications in transdermal hormone delivery. The authors thoroughly discussed the trends, advances, and challenges of the translation of microneedles from laboratory to clinical settings [36]. In another review, Ali and coworkers discussed the anatomy and biomechanical properties of the skin in association with microneedle insertion and drug permeation. The review also covered drug permeation modelling and the clinical translation of microneedles [37].

Transdermal delivery has been enhanced significantly by microneedle application to expand the range of potential transdermal candidates, capturing small molecules [38,39], macromolecules [40,41,42,43], cosmeceuticals [44,45,46], and particulate systems [47,48,49]. In most cases, microneedles may be used to transport molecules of any size or molecular weight. Various microneedle systems have been fabricated, each having its unique geometry, size, design, layout, density, composition, and materials. Microneedles may be constructed from a variety of different materials, such as glass, sugar, metal, silicon, ceramics, and polymers. Each category contains many specific materials which fulfil the requirements for microneedle production (i.e., mechanical strength, biocompatibility, and safety). Among these, safe, biodegradable, and biocompatible polymers emerged as promising options, and have received much attention and interest [50]. Polymers are suitable materials to fabricate all types of microneedles (i.e., dissolving, swelling, solid, coated, and hollow microneedles). Commonly used polymers include SU-8 photoresist, cyclic-olefin copolymer, polycarbonate, poly (methylmetha-acrylate), poly-lactic-co-glycolic acid (PLGA), poly-glycolic acid, polystyrene, polylactic acid, poly (vinyl pyrrolidone), polyvinyl alcohol, and sodium carboxy methyl cellulose. PLGA, chitosan, and hyaluronic acid were the most frequently used in microneedle fabrication [26,51,52]. Several research groups have recently evaluated various materials (i.e., natural, synthetic, semisynthetic polymers, and particle composites) for microneedle fabrication [53,54,55]. Microneedles made from natural materials receive substantial attention due to their excellent compatibility and minimal skin irritation [56,57]. Dabholkar summarized the use of natural materials (i.e., polysaccharides, polypeptides, and proteins) to produce biodegradable microneedles [56]. These carbohydrate materials include cellulose and derivatives, starch, and complex carbohydrate polymers (i.e., chitosan, alginates, pullulan, chondroitin sulfate, chitin, xanthan gum, and hyaluronic acid). Examples of protein polymers encompass gelatin, zein, fish scale, collagen, and silk fibroin. Damiri et al. also systemically discussed various carbohydrates for microneedle fabrication [58].

**Figure 2:**
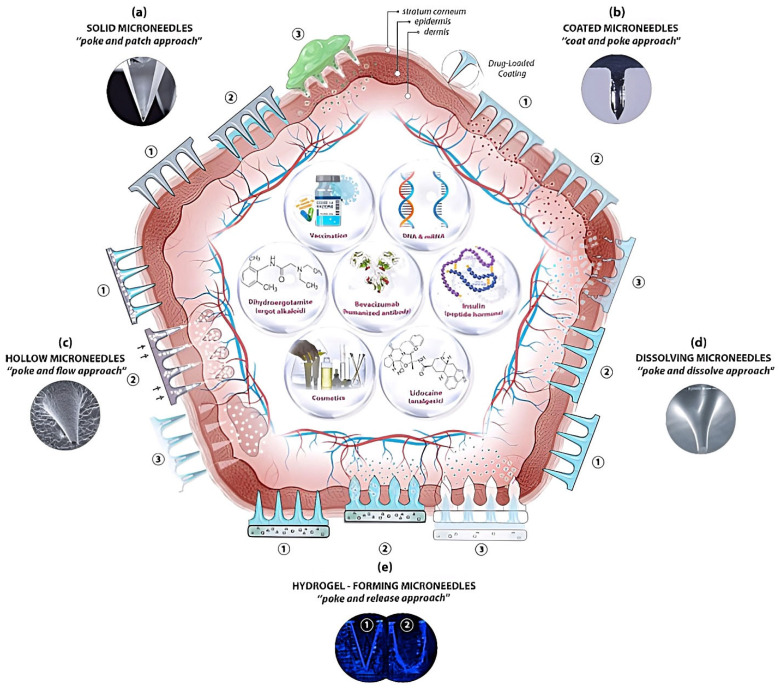
Schematic representation of microneedle-mediated transdermal drug delivery: (**a**) Solid microneedles, by creating transient hydrophilic microchannels in the skin, improve the drug permeation. (**b**) Drugs are coated onto the microneedle surface and dissolve quickly once inserted into the skin. (**c**) Hollow microneedles penetrate the skin, allowing the injection of the drug solution. (**d**) Upon skin insertion, dissolving microneedles dissolve and release the drug payload into the skin layers. (**e**) Swelling microneedles absorb interstitial skin fluid and swell to enhance drug diffusion through the porous swollen structure. Images reprinted with permission from [69].

**Figure 3:**
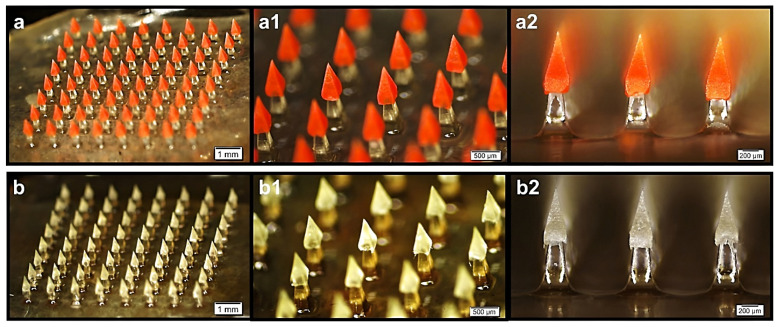
Microscopic images of chitosan-poly(L-lactide-co-D, L-lactide) microneedle array loaded with (**a**,**a1**,**a2**) rhodamine B dextran and (**b**,**b1**,**b2**) ovalbumin. Images reprinted with permission from [70].

Five types of microneedles have been employed in transdermal drug delivery, namely solid, hollow, coated, dissolving, and swelling microneedles (Figure 2). Microscopic images of dissolving microneedles are presented in Figure 3. Researchers in academic institutions and industrial companies have developed numerous methods for microneedle fabrication on different scales. Several reviews on the fabrication techniques of microneedles can be found in the scientific literature [31,53,54,59]. Microneedle fabrication methods include microelectromechanical systems, micromolding technique, additive manufacturing (i.e., fused deposition modelling, stereolithography, digital light processing, photon polymerization), atomized spraying technique, X-ray technique, laser technique (i.e., laser cutting, laser ablation), droplet-born air blowing, drawing lithography, pulling pipettes, and micro-injection molding. Among these methods, micromolding is the most frequently employed technique to produce microneedles in academic and industrial settings [43,52,60]. Microneedle-coating techniques include immersion coating, dip-coating method, layer-by-layer coating, drop-coating method, spray coating, electrohydrodynamic atomization, gas-jet drying, and piezoelectric inkjet printing. Recently, Ali et al. summarized common techniques for the production of dissolving microneedles, including micromolding, drawing lithography (i.e., thermal drawing, electro-drawing, magnetorheological drawing lithography), and additive manufacturing (3D printing) [53]. Notably, 3D printing has captured great interest as a promising technique for microneedle fabrication [61,62,63].

The fabricated microneedles were thoroughly characterized in various studies. Investigators examined microneedle formulations (i.e., drug solubility, drug-excipient compatibility, and rheological and interfacial properties), microneedle geometry and morphology (pre-insertion and post-insertion), mechanical properties (i.e., axial force, transverse force, base strength, and skin penetration force), microneedle dissolution, drug release, drug-loading capacity, drug distribution, skin penetration efficiency, safety (i.e., biological safety, skin irritation, and skin recovery), and physicochemical stability (i.e., hygroscopicity, swelling behavior, stability, water content, and solid state).

Microneedle insertion generates numerous microchannels in the skin [64,65]. Characterization of various aspects of microchannels confirms the successful skin microporation of microneedles. The characterization studies include morphology evaluation, skin resistance measurement, transepidermal water loss measurement, histological analysis, dye binding studies, microchannel depth (confocal laser scanning microscopy and optical coherence tomography), pore uniformity, and pore closure kinetics. After microneedle insertion, the created pores gradually close due to the skin viscoelasticity and the skin’s natural healing process. Several research groups have studied pore closure kinetics. Pore resealing could affect skin irritation and infection risk. Researchers reported a significant influence of pore closure on microneedle-mediated drug delivery [23,66,67]. The duration of pore closure ranged from a few hours to 72 h, depending on the skin types (animal and human skin), design of the experiment (in vitro, in vivo, and clinical studies), occlusion, microneedle dimensions, and formulation pH [22,23,67]. Haridass and colleagues reported that pores created by microneedle insertion (Nanopatch^®^) closed by 25% within 30 min and about 100% by 6h. Therefore, microneedle-formed pores are temporary and reversible, leading to rapid skin recovery within 1–2 days [68].

Several benefits and drawbacks of microneedles have been discussed in the scientific literature. Microneedles resolve all hypodermic needle-related issues: needlestick injuries, needle phobia, sharp waste, and transmission of blood-borne pathogens. The painless and noninvasive features of microneedle treatment enhance patients’ acceptability and compliance. Furthermore, microneedles improve drug bioavailability by bypassing first-pass hepatic metabolism and avoiding enzymatic degradation. Microneedles could provide a dose-sparing effect and robust immunological response for vaccines. The temporary and reversible skin disruption caused by microneedle insertion reduces the risk of skin irritation and infection. Moreover, safe, biocompatible, and biodegradable materials minimize the risk of inflammation reactions and biosafety issues. No requirement for healthcare professionals allows self-administration. The use of microneedles leads to a reduction in drug/vaccine wastage, no sharp waste and no risk of disease transmission. Microneedle design could be customized to achieve sustained or immediate drug release. The needle length and penetration depth could be altered to enhance drug delivery to targeted skin layers. The dimensions and designs of the microneedle array could be modified to achieve a large skin area treatment. Interestingly, microneedles enable transdermal delivery of multiple drugs simultaneously. Individualized therapy and drug delivery to the specific patient population could be achieved by adjusting microneedles’ geometry, dimensions, designs, and materials. Microneedle manufacturing should be efficient, reproducible, and low-cost. Furthermore, improved drug stability in the solid form of microneedles negates the requirement for cold-chain storage and transportation. Besides, combining microneedles with other physical enhancement technologies facilitates synergistic, enhanced drug-delivery effects.

Several disadvantages of microneedles have been noted. Microneedles could carry a very limited dose of drug-loaded quantity. Polymeric microneedles might have insufficient robustness and weak mechanical properties to successfully penetrate the skin. Furthermore, skin viscoelasticity reduces microneedles’ penetration depth. Skin variables (thickness, hydration level, viscoelastic properties) create a challenge to achieve consistent skin penetration depth. Microneedles could be fabricated from only a range of appropriate materials. Harsh manufacturing conditions could cause the degradation of thermolabile drugs. Blockage of hollow microneedle bore reduces the needle penetration and drug delivery efficiency. Moreover, there is a lack of regulatory guidance, current good manufacturing practices, and standardized quality control systems for large-scale manufacturing. The shortage of investment from the pharmaceutical industry is also an obstacle to the development of microneedle products. 

### 1.4. Introduction of Biopharmaceuticals

Biopharmaceuticals have long been regarded as safe and effective therapeutics [71] with excellent efficacy and minimal risk of adverse effects or toxicity [72,73]. Their complicated structure enhances their functional specificity to the therapeutic target. As opposed to conventional small molecules, these biomolecules are highly potent, thus requiring a low treatment dose and minimizing any safety issues [41,72]. Several biomolecules could be administered to directly replace the defective endogenous proteins. These macromolecules could alleviate and cure symptoms of a variety of diseases such as inflammation, neurodegenerative conditions, genetic disorders, cancer therapy, immunization, genetic disorders, infectious diseases, rheumatoid arthritis, osteoporosis, and diabetes, among many others [74,75]. Interestingly, immunotherapy captures most of the applications. These biomolecules could function as enzymes, immunostimulators, cellular regulators, or molecular transporters, thus serving a critical role in bodily activities [76,77,78]. Recent advances in biotechnology, especially recombinant DNA technology, have enabled the production of numerous biopharmaceutical products. Researchers anticipated that the development of such products would grow tremendously.

The literature has revealed several drawbacks of biopharmaceutical molecules. Their large molecular weight and size, as well as hydrophilic properties, hinder their absorption into biological membranes [41]. Moreover, these molecules are unstable, losing most of their bioactivity when exposed to conditions such as moisture, elevated temperature, or proteolytic enzymes. In addition, their short half-lives, attributed to rapid degradation by metabolic enzymes, lead to frequent administration and inconvenience. Biomolecules could cause some severe adverse effects, including autoimmunities or non-specific inflammatory responses [79]. Additionally, their thermosensitivity and low stability create obstacles to formulation development and production processes.

### 1.5. Current Status of Biopharmaceuticals

Ever since the first successful launch of Humulin (a recombinant human insulin) 30 years ago, the development of numerous biopharmaceutical products (i.e., peptides, enzymes, monoclonal antibodies, proteins, and biologics) has been the primary driving force of the pharmaceutical industry [73]. To date, the US FDA has approved more than 100 recombinant proteins, while several products are currently undergoing various phases of clinical trials [80]. For instance, Semaglutide (a glucagon-like peptide-1 receptor agonist) received FDA approval as a diabetes therapy in 2019 under the brand Rybelsus™. The global market of biopharmaceutical products is anticipated to reach USD 388 billion by 2024 and grow exponentially in the following years due to the considerable potential and widespread interest. Notably, over half of the top 20 blockbuster drugs fall into the biopharmaceutical category [81]. The enhanced efficiency of protein expression and synthesis contributes to the rapid advancement of the biopharmaceutical field.

### 1.6. Conventional Parenteral Administration

Due to their unique physicochemical properties, biomolecules could be administered using a few viable drug delivery routes, including intravenous, transdermal, intravesical, nasal, ocular, and rectal routes [82]. Among these, parenteral administration (i.e., intravenous, subcutaneous, and intramuscular) appears most effective for biopharmaceutical products [72,83]. Typically, conventional parenteral injection provides a low-cost platform for rapid drug delivery and high bioavailability [81,84].

Nevertheless, traditional injections using hypodermic needles carry several limitations. Evidently, this method generates pain, needle anxiety, needlestick injuries, and risks of disease transmission (i.e., hepatitis B and C [41]), hence compromising patient compliance and acceptability [81]. Specifically, those with chronic diseases (i.e., rheumatoid arthritis and diabetes) consider hypodermic injection uncomfortable and inconvenient. Furthermore, frequent injections can cause some complications, such as phlebitis, tissue necrosis, and the possibility of adverse effects [85,86]. Moreover, patients would always require trained healthcare providers to deliver the dosage. The Centers for Disease Control and Prevention (CDC) estimates that 385,000 healthcare workers in the United States suffer needlestick injuries every year, exposing them to the risk of contracting and spreading diseases. Moreover, proteases, opsonization, fast metabolism, and agglutination could cause significant instability of biomolecules in the systemic circulation [87,88]. Several techniques have been employed to improve the stability of biomolecules, such as chemical modification, colloidal delivery systems, thermosensitive gels, and polymeric nanotechnology-based systems [89,90].

### 1.7. Transdermal Delivery of Biopharmaceuticals

To circumvent the injection-associated drawbacks, a novel delivery strategy is expected to improve drug stability and enhance therapeutic efficacy. In particular, the transdermal delivery system has recently emerged as a potential administration platform for biomolecules [72]. The molecules’ physicochemical properties (i.e., shape, size, molecular weight, solubility, melting point, ionization, and hydrophilicity), the features of the delivery system, and skin properties (i.e., age, temperature, gender, structure, and disease conditions) govern the safety and efficacy of transdermal biopharmaceutical delivery systems. In addition to the typical benefits of transdermal delivery systems, the feature of sustained drug delivery is especially beneficial for molecules with short half-lives and frequent dosing. Skin delivery involves minimal proteolytic and enzymatic degradation, markedly lower than mucosal or oral routes [72], thus improving the therapeutic efficacy of biopharmaceutical molecules. In 2022, Zhang presented a comprehensive review of various strategies (applications and mechanisms) to enhance the transdermal delivery of biopharmaceutical compounds. In particular, the authors emphasized the prevalent use of penetration enhancers, nanovesicles, and microneedles [91].

However, researchers have reported several drawbacks of the transdermal delivery system for macromolecules. The physicochemical properties of these molecules (i.e., high molecular weight and hydrophilicity) go against the Lipinski rules governing effective drug delivery across the skin [83]. Consequently, the extent of passive permeation of these biomolecules is negligible. Several macromolecules could interact with the components of the stratum corneum at varying degrees, altering the rate and extent of the drug permeation. Furthermore, transdermal delivery of biomolecules could induce skin irritation or local inflammation. Several innovative technologies have been developed and employed to disrupt the skin structure, especially the stratum corneum layer, to enhance the transdermal delivery of macromolecules. An ideal strategy should minimize drug degradation and protect drug structural integrity during production, distribution, and usage [92]. Significantly, the most common and effective methods for enhanced transdermal delivery of biomolecules are thermal ablation and microneedles.

### 1.8. Microneedles for Biopharmaceutical Delivery

Numerous in vitro, in vivo, and clinical investigations have revealed the application of microneedles in enhancing the transdermal delivery of macromolecules [93,94,95,96]. Aich et al. reviewed several studies on microneedles for the transdermal delivery of biomolecules (i.e., proteins and peptides). The authors discussed various designs, types, formulations, fabrication methods, advantages, and disadvantages of microneedles [97]. These micron-sized needles porate the skin layers to create transient microchannels in the skin, which function as diffusion pathways for biomolecules to reach deeper skin layers. With the stratum corneum disrupted and microchannels formed, hydrophilic and large molecules may be rapidly transported through the skin and into the systemic circulation [98]. In general, microneedles could efficiently carry and deliver therapeutic agents of any size into the skin. The drug-loading capacity of microneedles depends on the needles’ dimensions, designs, geometries, and densities, as well as the drug formulation [99]. The microneedle materials could be tailored to achieve low-cost production, improved penetration depth, and customizable drug delivery or release kinetics (i.e., bolus or sustained drug release) [100,101]. Microneedles could carry drug-encapsulated micro/nanoparticles to deliver the drug across the skin for an extended period. These particulate systems include nanoparticles [24], nanomicelles [102,103], and mesoporous silica particles [104], among many others. In 2022, Oh and coworkers provided a comprehensive review of nanoparticle-integrated microneedles for the sustained release and delivery of macromolecules [105]. Several review articles have discussed the strategies of particle-integrated microneedles for transdermal delivery [28,106]. Different microneedle types (i.e., solid, hollow, coated, dissolving, and swelling microneedles) substantially enhance the transdermal delivery of small molecules, macromolecules, and particle systems. Microneedle-mediated delivery is effective for various biomolecules, such as insulin, etanercept, growth hormone, erythropoietin, glucagon, parathyroid hormone, desmopressin, lysozyme, bovine serum albumin, human immunoglobulin A, and oligonucleotides [78,100,107,108]. Immunotherapy using monoclonal antibodies has benefited from microneedle application to control the immune response. Microneedle treatment provides a viable alternative to the current painful and inconvenient injection of insulin [109]. Furthermore, microneedles reduce the possibility of protein denaturation, thus expanding the transdermal field to encompass these ‘difficult’ biomolecules. The needles’ polymeric structure effectively encapsulates and protects these molecules. Additionally, the drug-loaded quantity could be increased to a certain extent by optimizing the microneedle design and drug formulation. Mild conditions in microneedle production and the dry, solid form of the product enhance the drug’s stability and preserve its bioactivity; this is especially critical for thermosensitive biomolecules. Moreover, the inclusion of stabilizers (i.e., trehalose or mannitol) into the drug formulation could further improve the product’s stability and efficacy [110]. Interestingly, microneedles could deliver drugs locally into targeted skin regions, rather than driving the drug into the blood circulation. This feature mitigates self-reactive T-cell overstimulation, avoids immune depletion, and lessens the risk of immune side effects [111].

Even though microneedles offer several advantages for the transdermal delivery of biomolecules, this technology poses certain shortcomings. Specifically, the two-step application of solid microneedles in the “poke and patch” strategy can lead to erroneous dosing [112]. Furthermore, a limited drug-loading capacity is a particular issue with coated microneedles, while needle bore blockage and drug leakage are common challenges for hollow microneedle design. Drug injection or infusion via hollow microneedles would require experienced healthcare providers and a complicated system setup [113,114]. Mechanical robustness and needle sharpness are critical quality attributes of dissolving microneedles, which have to be optimized to ensure product performance [115]. To minimize any enzymatic degradation of biomolecules in the skin tissue, microneedles’ geometries and dimensions should be fine-tuned to shorten the drug diffusion path and place the drug in the targeted delivery site. In many cases, drug encapsulation into the needle polymeric structure is inadequate to completely preserve the drug bioactivity, thus causing some risks of partial drug degradation during production or application. Highly sensitive macromolecules could be loaded into some particulate systems (i.e., microparticles, nanoparticles, liposomes, etc.) before encapsulation into the needle polymeric matrix [116].

### 1.9. Stability of Biopharmaceuticals in Microneedles

Biopharmaceutical drugs are generally more susceptible to degradation by extreme conditions (i.e., pH, temperature, and humidity) than small molecules [117]. Organic solvents, for instance, dichloromethane, ethyl acetate, and dimethyl carbonate, promote protein breakdown. When exposed to water, biopharmaceutical drugs often experience aggregation, denaturation, and precipitation [118]. Structural modification of biomolecules might cause unintended consequences, such as a reduction in drug efficacy, loss of bioactivity, compromised drug safety profile, and risks of unexpected immunogenicity. Any formulation development strategies should be developed with an in-depth understanding of the biomolecules’ physicochemical properties and stability aspects. Optimal production processes, material selection, and formulation development should ensure the drug’s integrity, stability, and efficacy [78,119,120]. The degradation mechanisms and factors affecting drug stability in different microneedle types are presented in Table 1.

The increased thermostability of biomolecules encapsulated in microneedle structure attracts substantial interest, since these molecules lose their bioactivity and efficacy when stored at ambient conditions in liquid or lyophilized formulations. The skin, as a robust immunological organ, makes protein immunogenicity a major safety concern. Therefore, researchers have highlighted the necessity to meticulously characterize protein aggregates and subvisible particles released from microneedle products [121]. The use of high temperature, a vacuum, centrifugation, organic solvents, pH, or UV light exposure in the conventional microneedle manufacturing processes may impose some harmful effects on drug stability [122,123,124]. Therefore, microneedle production should employ low temperatures and limited use of organic solvents [122,125,126,127]. In a comprehensive review article, Maaden et al. presented potential causes of biomolecule degradation during each stage of microneedle production, storage, and application [32].

Several investigations in the scientific literature have revealed the enhanced stability of various biomolecules loaded in microneedle structures [128,129,130]. For example, insulin was loaded into dissolving microneedles for enhanced transdermal delivery. This strategy allowed the preservation of insulin’s functional activity for one month at varying temperatures (−80 to 40 °C) [131]. Similarly, when insulin was embedded in starch and gelatin microneedles, the drug was stable at ambient temperature or higher for at least one month of storage [132]. Dissolving microneedles containing insulin were produced by Migalska and colleagues, who uncovered no evidence of chemical or secondary structural alterations in denatured insulin [122]. Kochhar and colleagues reported that bovine serum albumin was stable under UV light exposure (low intensity of 11.0 W/sq.cm and short duration of 3.5 s) during the fabrication process (photolithography) of microneedles [133,134]. Human growth hormone activity was also maintained after being encapsulated inside dissolving microneedles and stored in room conditions for up to 15 months [100]. The encapsulation of immunoglobulin G into hyaluronan microneedles improved drug stability. These drug-loaded dissolving microneedles could effectively porate the skin, dissolve rapidly, and release the drug payload. The researchers studied the drug’s stability and aggregation on molecular, submicron, and micron scales [135]. A study conducted by Hiraishi and coworkers demonstrated that the environmental humidity significantly affected the needles’ mechanical properties and protein stability. The needle robustness was inversely correlated with the surrounding humidity, as shown by the mechanical failure force experiment. Moreover, proteins were unstable in humid conditions, which caused their unfolding, aggregation, and chemical degradation [136]. Park and colleagues fabricated BSA-loaded dissolving microneedles using a micromolding technique in which the drug formulation was cast on the mold with a molten polymer (135 °C). Dynamic light scattering study indicated no major change in BSA structure after exposure to 135 °C for 10 min; however, an increase in the heating duration to 20 and 30 min led to protein aggregation. Notably, BSA was completely denatured after one hour in the molten polymer at 135 °C. Collectively, the use of high temperatures is unfit for macromolecules [137]. Lee and colleagues fabricated dissolving microneedles carrying BSA and lysozyme at room temperature. The authors evaluated the structural and functional features of lysozyme and reported no substantial degradation of the drug after two-month storage at room temperature and humidity [138]. In another investigation, Fukushima and coworkers employed an enzyme immunoassay and LC/MS/MS analysis to confirm the one-month stability of rhGH loaded in dissolving microneedles [126]. Ameri and colleagues studied the stability of parathyroid hormone (PTH) when coated onto solid microneedles. Oxidation and aggregation were the major degradation mechanisms of coated PTH. The inclusion of sucrose in the coating composition led to a significant reduction in PTH aggregation (from 7% to 0.5%). In particular, oxidation and aggregation accounted for 1% and 7% of drug degradation, respectively. Furthermore, metal elements present in excipients and metal microneedles catalyzed and accelerated the drug oxidation process. Importantly, PTH bioactivity was preserved for up to 18 months when kept at ambient temperature and 60% relative humidity [139]. Similarly, the encapsulation of oxytocin in dissolving microneedles significantly improved the drug stability. The product was stable after two-month storage at 40 °C/75%RH. The addition of trehalose to the microneedle formulation further stabilized the drug: 75% of the drug remained stable after 12 months at 40 °C [140]. Further research on drug stability, drug encapsulation, mechanical properties, and the safety of microneedles will expand microneedle application for the effective transdermal delivery of biomolecules.

## 2. Microneedle Types for Biopharmaceutical Delivery

### 2.1. Solid Microneedles

Solid microneedles generally require two steps to administer drugs. Solid microneedles are first inserted into the skin and subsequently removed, leaving behind transient hydrophilic microchannels. After that, a drug-loaded topical formulation (i.e., gel, cream, lotion, ointment) or a transdermal patch is applied over the microchannels to deliver the drug (Table 2) [65,108,141,142]. The microchannels produced by the solid microneedle insertion allow the applied drugs to diffuse passively into the skin layers (Figure 2a). After the application of the drug formulation on the treated site, the drug delivery through microneedle-created microchannels could continue until the drug is depleted or the channels are closed. The dimensions, geometry, sharpness, and density of the microneedles utilized for skin pretreatment affect drug transport into and across the skin [143,144]. In addition, the physicochemical properties and molecular weight of the drugs also significantly impact the efficiency of microneedle-assisted delivery [45].

Solid microneedles could be fabricated from various materials, such as glass, metal, silicon, and polymers. The common designs of solid microneedles include solid array, flexible patch, or roller type. Numerous biomolecules have benefited from skin disruption by solid microneedle pretreatment. A number of investigations have focused on the microneedle-mediated delivery of fluorescein isothiocyanate-labelled ovalbumin and insulin, ovalbumin-conjugated nanoparticles, human immunoglobulin G, calcein, bovine serum albumin, fluorescein isothiocyanate-coupled dextran, melanostatin, rigin, palmitoyl-pentapeptide, and genes [45,145,146]. In general, microneedle treatment substantially enhances the intradermal and transdermal delivery of most macromolecules. Furthermore, the enhancement of drug delivery is inversely correlated with the drug’s molecular weight [45,147].

Several research groups studied the efficiency of solid microneedle treatment for the intradermal delivery of insulin (in vitro and in vivo) and reported a significant reduction in blood glucose levels [148,149]. Martanto and colleagues revealed an 80% decrease in the blood glucose levels in diabetic rats due to solid microneedle insertion. Furthermore, these microneedles improved the delivery of insulin to a comparable level of 0.05–0.5 units of insulin administered by subcutaneous injection [150]. Interestingly, Qiu and coworkers designed an insulin-loaded lyophilized hydrogel patch to provide sustained and continuous drug delivery through microneedle-formed channels in the skin for at least eight hours. This novel formulation provided a markedly longer duration of effects than the conventional subcutaneous injection. Moreover, insulin could retain 90% of its bioactivity after six-month storage at 4 °C [151].

### 2.2. Coated Microneedles

An improved strategy to employ solid microneedles in enhancing transdermal drug delivery is to coat drug formulations onto the needle surface (Table 2). Several coating methods (i.e., dip coating, casting, and deposition [152,153]) have been developed and evaluated for coating drug formulations onto the needle surface. Once inserted into the skin, the coating layer disintegrates and dissolves rapidly, depositing the drug into the targeted skin layers [110] (Figure 2b). Compared to the two-step application process of solid microneedles, this single-step technique (coated microneedles) is remarkably more efficient, controlled, and convenient. Notably, most in vivo studies of transdermal macromolecule delivery have used coated microneedles. Unfortunately, coated microneedles could carry only a very small quantity of drug on their limited surface. Additionally, an excessive coating may result in compromised microneedles’ mechanical strength and sharpness. Thus, coated microneedles benefit highly potent molecules, which require a relatively low therapeutic dose, such as desmopressin, human growth hormone, interferon alpha, and most macromolecules [154,155]. Researchers must endeavor to optimize the coating process and formulation to achieve an accurate, reliable, and reproducible quantity of drugs coated on the needles.

Several macromolecules could penetrate the skin effectively with the application of coated microneedles. These biopharmaceutical drugs include desmopressin, bovine serum albumin, interferon-alpha, parathyroid hormone, peptide A, insulin, recombinant human erythropoietin alfa, bovine pancreatic ribonuclease A, antisense oligonucleotides, erythropoietin, ovalbumin, and human growth hormone [154,155,156,157,158,159,160,161]. Notably, coated microneedles could deliver hydrophobic peptides into human skin in vitro and mouse skin in vivo [162]. Li and associates coated metal microneedles with different molecules (proteins, immiscible molecules, and nanoparticles) to deliver multiple therapies from a single microneedle array [163]. The bioavailability of human growth hormone and peptide A coated on solid microneedles was equivalent to that of subcutaneous injections, thus demonstrating the efficiency of coated microneedles in transdermal drug delivery [154,158].

Some noticeable coated microneedle systems are the Macroflux^®^ microneedle array (titanium microneedles) and 3M solid microstructured transdermal system (sMTS). The Macroflux^®^ system could coat various biomolecules (i.e., biologics, peptides, proteins, and vaccines) onto the solid microneedle surface. Among these, parathyroid hormone 1-34 (PTH 1-34), a medication for postmenopausal osteoporosis treatment, has received a great deal of attention in preclinical and clinical trials [157]. Importantly, PTH remained stable in the finished product after two-year storage at 25 °C, thus eliminating any cold-chain or special storage requirements. The insertion of PTH-coated microneedles led to an abrupt increase in the drug plasma level, with the T_max_ three-fold faster than the control FORTEO^®^ subcutaneous injection [139]. Similarly, Macroflux^®^ desmopressin-coated microneedles provided rapid drug delivery in vivo and a therapeutic dose for antidiuretic effects without any pain or skin irritation [164]. Furthermore, the 3M sMTS (coated microneedles) could carry a drug payload of up to 0.3 mg. Peptide A became significantly more stable after being coated on the sMTS [158].

### 2.3. Hollow Microneedles

In a nutshell, hollow microneedles are downscaled hypodermic needles in micron size with a similar configuration (Table 2). Hollow microneedles allow the injection or infusion of a drug solution into the skin layers (i.e., epidermis or dermis) at a controlled rate and in a non-invasive way [165] (Figure 2c). The simplest route of drug transport via hollow microneedles is passive diffusion. Given the slow passive drug permeation into the dense skin tissue, researchers have applied a certain level of pressure to facilitate the drug delivery [7]. A noted advantage of hollow microneedles lies in their capacity to deliver a large and accurate quantity of drugs into the skin [6,166]. An optimized microneedle design should possess an acceptable mechanical strength to avoid needle breakage during skin insertion and minimize the risk of bore blockage—a major issue of hollow microneedles. Scientists have designed hollow microneedles with off-centered bores on the side of the tips to prevent bore blockage and expose the drug to surrounding skin tissue. The scientific literature reveals the in vivo application of hollow microneedles in improving the transdermal delivery of various macromolecules, such as proteins, peptides, oligonucleotides, and vaccines.

A commonly used application of hollow microneedles is to administer insulin in a painless and noninvasive “poke and flow” technique [122,148,167]. Researchers have investigated transdermal insulin delivery with hollow microneedles in in vitro, in vivo, and clinical studies. The drug, loaded in a liquid dispenser or a reservoir, was driven into the skin using passive diffusion, pressure, electrical assistance, or compressed CO_2_. In general, intradermal delivery of insulin via hollow microneedles provided a faster absorption rate and a superior treatment efficacy than the traditional subcutaneous injection [168]. Interestingly, a partial retraction of hollow microneedles (approximately 200 μm) allowed the injection of a significantly larger volume of drug solution. Moreover, a study on children with type 1 diabetes revealed that insulin injection via hollow microneedles resulted in faster healing and less pain than conventional injection methods [169]. McAllister and coworkers reported that at 10 psi pressure, a single glass microneedle inserted into the skin of a hairless rat for 30 min could deliver 32 μL insulin solution [170]. Notably, Xenikakis et al. developed two designs of hollow microneedles using 3D printing and liquid crystal display methods. The researchers characterized the needle dimensions using scanning electron microscopy, the volumetric properties of microneedles and microchannels using microfocus computed tomography, and the mechanical properties and skin penetration efficiency using finite element analysis simulation. The fabricated hollow microneedles facilitated insulin delivery across human skin in vitro [171].

Furthermore, hollow microneedles could enhance transdermal delivery of various macromolecules, such as β-galactosidase, formaldehyde-inactivated botulinum toxoid [172], synthetic mRNA [173], cascade blue, dextran-cascade blue, FITC-dextran [174], human growth hormone, equine tetanus antitoxin [175], and ovalbumin-loaded PLGA nanoparticles [47]. 3M has introduced a hollow microstructured transdermal system (hMTS) to inject liquid formulations into the skin. In this system, hollow microneedles are attached to a glass cartridge. This spring-controlled device enables the self-injection of up to 1.5 mL drug solution. In particular, the 3M™ hMTS device could effectively deliver equine tetanus antitoxin and human growth hormone into the skin in vivo. The researchers reported comparable pharmacokinetic profiles of these drugs in domestic swine when delivered via hMTS or subcutaneous injection [175].

### 2.4. Dissolving Microneedles

A novel design of microneedles—dissolving microneedles—has been receiving substantial interest from the academic and industrial sectors (Table 2). Recently, Ali and coworkers provided a comprehensive review of dissolving microneedles (especially designs and materials) for the transdermal delivery of various macromolecules [53]. These needles carry the therapeutic agents inside their polymeric matrix [29]. Upon skin insertion, these drug-loaded microneedles disintegrate and dissolve in the interstitial skin fluid to release the drug payload (Figure 2d). This system could provide bolus or sustained drug release kinetics, depending on the dissolution rate of the polymeric materials and the microneedle application duration [176,177,178,179,180]. The primary concern of dissolving microneedles is their mechanical robustness, which has an inverse correlation with the drug-loaded quantity. Furthermore, the physicochemical properties of the materials and microneedle design parameters also markedly impact the needles’ mechanical strength and drug release kinetics [181]. The aspect ratio of microneedle length to base dimensions directly influences the needle robustness [182]. Most recent research on dissolving microneedles has emphasized the design and geometries of microneedles [4,27,29,183,184]. Several novel microneedle designs have been proposed and evaluated for effective skin penetration and drug delivery [185]. Suitable biodegradable and water-soluble polymers for dissolving microneedles include carboxymethylcellulose [100], maltose [186], chitosan [60,187,188], polyvinyl alcohol (PVA) [189], hyaluronic acid, and polyvinylpyrrolidone. Furthermore, mild manufacturing conditions are preferable to improve the stability and protect the bioactivity of biopharmaceutical drugs [53,190].

Investigators have revealed that dissolving microneedles could effectively deliver insulin into the skin, thereby reducing the blood glucose levels in mice, diabetic rats, and dogs [191,192]. Specifically, a glucose-responsive microneedle-mediated transdermal delivery of insulin received significant interest [193,194]. In particular, the pharmacokinetic profile of insulin was comparable between microneedle treatment and subcutaneous injection [195]. Furthermore, the encapsulation of insulin into dissolving microneedles improved the drug stability, bioactivity, and bioavailability [195]. For instance, when encapsulated into starch and gelatin microneedles, insulin could retain over 90% of its bioavailability after one-month storage at 25 or 37 °C [132]. Jung and colleagues employed a mild droplet-born air-blowing technique to produce insulin-loaded microneedles with a relative bioavailability of 96.6% [196]. Yu and coworkers developed a “smart insulin patch” with a crosslinked hyaluronic acid matrix containing glucose-responsive vesicles to effectively and rapidly lower the blood glucose levels in diabetic mice [101]. Similarly, Yang and associates developed a glucose-responsive closed-loop system for transdermal delivery of insulin and glucagon. The release of insulin and glucagon could be adjusted automatically by the change in the blood glucose levels. The researchers demonstrated the long-term effectiveness of this microneedle system on mice and minipigs with induced type 1 diabetes [197]. Demir et al. employed the combination of gelatin methacrylate, polyethylene glycol diacrylate, and MoS2 nanosheets to fabricate polymeric microneedles with desired drug release kinetics. The MoS2 needles could penetrate mice and porcine skin and release insulin in ex vivo and in vivo studies. Furthermore, the microneedle-induced level of blood glucose reduction was equivalent to subcutaneous injection in mice and pigs [198].

**Figure 4:**
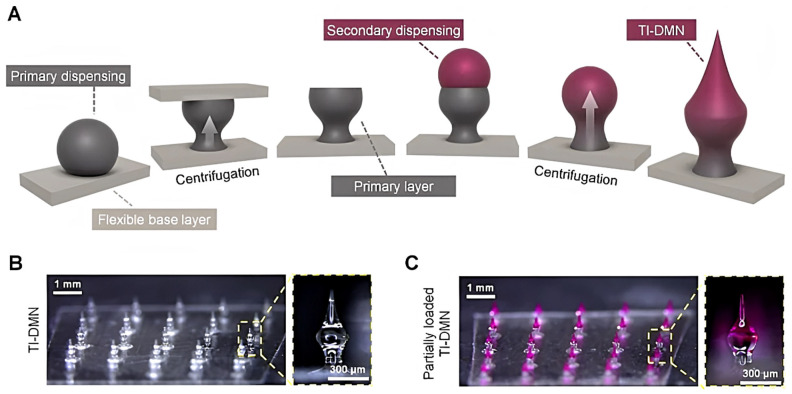
Schematic representation of hyaluronic acid-based tissue-interlocking dissolving microneedles. (**A**) Fabrication steps, (**B**) microscopic images, (**C**) microneedle partially loaded with Rhodamine B. Images reprinted with permission from [210].

Dissolving microneedles function as a carrier to transdermally deliver numerous biopharmaceutical agents, such as calcein, bovine serum albumin, immunoglobulin G [135], cyclosporin A [199], fluorescein isothiocyanate-labelled dextran [200], interferon-α-2b [201], polymyxin B [202], lysozyme [203,204], FITC-BSA [205], glucagon [206], human parathyroid hormone [207], vascular endothelial growth factor [208], monoclonal IgG [135], rhGH, desmopressin [126], and leuprolide acetate [209]. Fakhraei Lahiji et al. developed a novel hyaluronic acid-based tissue-interlocking microneedle to improve needle-to-skin adhesion, thus increasing transdermal delivery of various biomolecules (Figure 4) [210].

Interferon-α-2b-loaded dissolving microneedles were bioequivalent to intramuscular injection, demonstrating that microneedles could be a reliable alternative to conventional intramuscular administration [201]. When Lahiji et al. fabricated lysozyme-loaded microneedles at 4 °C, dried the system at ambient temperature, and included stabilizing agents in the formulation, they obtained the drug bioactivity of 99% for 12 weeks [203]. Microneedles significantly improved the stability of parathyroid hormone compared to the control solution, while the drug bioavailability in microneedles was 100%. Consequently, microneedle therapy led to slower bone loss and increased bone density in rats [207]. Yao et al. reported that parathyroid hormone-loaded hydrogel microneedles could stimulate wound angiogenesis, tissue restoration, and collagen production, thus leading to considerably rapid wound healing of the skin [211]. Chitosan microneedles containing vascular endothelial growth factor promoted rapid collagen deposition, inflammatory reduction, and tissue regeneration in wound healing [208]. Chen and colleagues designed a microneedle array with chitosan dissolving microneedles (to provide rapid drug release), distributed on a poly(L-lactide-co-D, L-lactide) (PLA) base substrate (to provide mechanical strength for complete skin insertion). Upon skin penetration, the needles were detached from the array substrate and embedded in the skin tissue, thus enabling sustained drug delivery [70].

Chen et al. prepared dissolving polyvinylpyrrolidone-based microneedles with biphasic release kinetics of ofloxacin and basic fibroblast growth factor (bFGF) for wound healing. Rapid dissolution of the array base released ofloxacin to prohibit infection and then delivered bFGF-loaded PLGA microspheres to the wound areas. The gradual disintegration of the PLGA microspheres slowly released bFGF to enhance wound healing. Consequently, this microneedle system enabled rapid and effective wound healing in vivo [212]. In another study, Sim and associates developed bilayer teriparatide acetate-loaded dissolving microneedles using the centrifugal lithography technique. These microneedles contained the drug payload on the top layer and hyaluronic acid on the bottom layer. The addition of trehalose to the formulation considerably improved the drug stability. Furthermore, the needles rapidly delivered 87.6% of the drug into porcine skin after 5 min [213]. An investigation by Zhou et al. demonstrated the successful fabrication of dissolving microneedles from a natural material (Bletilla striata polysaccharide). These microneedles provided superior mechanical robustness and stability to hyaluronic acid and polyvinyl alcohol microneedles. The investigators reported excellent cell compatibility, minimal bacterial entry, no infection, and negligible skin irritation from these needles. Furthermore, a circular dichroism study revealed that ovalbumin remained stable within the needle structure for 21 days [57]. Similarly, GhavamiNejad and colleagues fabricated a hyaluronic acid-based dissolving microneedle patch to deliver a peptide transdermally (PRL-2903, somatostatin receptor type 2 antagonist). Notably, the researchers employed molecular dynamics simulations to evaluate the stabilizing effects of hyaluronic acid polymers on PRL-2903 structure. In vivo experiments demonstrated that PRL-2903-encapsulated microneedles markedly increased the glucagon level and recovered blood glucose levels, thus controlling hypoglycemia [214]. Interestingly, Hu and associates proposed a novel design for dissolving polyvinylpyrrolidone-based microneedles. The authors developed mechanically robust hyaluronidase-powered microneedles, which provided efficient skin poration and enhanced transdermal delivery of macromolecules. Hyaluronidase depolymerizes hyaluronic acid in the skin tissue to expand the subcutaneous space and disrupt the extracellular matrix barrier, thus improving drug permeation [215]. Panda et al. fabricated biodegradable and biocompatible microneedles from poly (D, L-lactic co-glycolic acid) (PLGA) and polyvinyl alcohol (PVA) using a mold-casting method. These needles could carry FITC-dextran (4 kDa) and effectively penetrate the skin to significantly enhance drug delivery ex vivo [216]. Men and coworkers developed recombinant hirudin-encapsulated dissolving microneedles for the treatment of thrombosis. The investigators fabricated the bilayer needles from the combination of polyvinylpyrrolidone and polyvinyl alcohol using a mold-casting method. After skin insertion, the needles rapidly dissolved, losing 78.67% of the needle length and releasing 68.12% of the drug payload. Both in vitro and in vivo studies revealed the efficacy of these microneedles [217]. Don and colleagues fabricated drug-loaded dissolving microneedles from a natural polymer (ulvan) using a casting technique. The authors reported that the needles could porate porcine skin in vitro and dissolved quickly in two minutes (90% reduction in the needle length) to release the drug payload (i.e., rhodamine 6G and bovine serum albumin–fluorescein isothiocyanate conjugate) into the skin tissue. Therefore, these needles significantly enhanced the transdermal drug delivery in vitro. Moreover, ulvan microneedles were biocompatible with HaCaT and NIH3T3 cells [218].

Notably, researchers have developed porous polymeric microneedles with microchannel networks to facilitate drug permeation [219,220,221]. In 2022, Tabassum et al. employed a combination of dry and wet etching techniques to develop novel porous silicon microneedles with controlled degradability, porosity, drug payload, and mechanical robustness. The researchers used electrochemical anodization to produce the conformal porous surface with customizable thickness, which dictated the biodegradable and mechanical properties of the needles. These microneedles could carry and deliver small molecules and biotherapeutics into and across porcine skin ex vivo [222].

Interestingly, several research groups have designed separable microneedle arrays for rapid drug release and short insertion duration [43,223,224,225]. Yang and coworkers developed separable dissolving microneedles from PAA/NaHCO_3_-silk protein for transdermal delivery of recombinant human growth hormone (rhGH). The mild fabrication process improved the stability and bioavailability of rhGH. This microneedle system could deliver the drug sustainably for seven days and provided a comparable effect to daily subcutaneous rhGH administration [226]. In another investigation, Li and colleagues fabricated separable thermosensitive hydrogel microneedles by crosslinking gelatin and carboxylic end-capped poly(*N*-isopropylacrylamide). These microneedles provided rapid separation and effective drug release within seconds. This microneedle system significantly enhanced the transdermal delivery of insulin across the skin of diabetic mice, providing a substantial hypoglycemic effect [227].

### 2.5. Swelling Microneedles

Hydrogel-forming swelling microneedles—the latest microneedle type—are generally fabricated from crosslinked polymeric materials [228]. When inserted into the skin, these needles quickly absorb interstitial skin fluid and swell, thus creating a swollen, porous structure, which serves as an unobstructed pathway for drug diffusion (Figure 2e). A noted benefit of this microneedle type is that the swollen microneedle structure can be removed from the skin intact, leaving behind a negligible polymeric residue in the skin. Typically, hydrogel-forming microneedles do not contain the drug within their structure; instead, the drug is loaded into a reservoir located on top of the microneedle array [229]. This feature eliminates the impact of drug-loading quantity on the needles’ mechanical properties and skin penetration efficiency. Moreover, the drug payload is independent of the needle dimensions, geometry, or surface area. Hence, this microneedle system enables transdermal delivery of a substantially large drug dose (Table 2).

Swelling microneedles have effectively delivered various biopharmaceutical drugs into the skin, such as bovine serum albumin [230], gap junction blocker (GAP-26) [231], insulin [232], bevacizumab [233], and ovalbumin [229]. Interestingly, Cao and associates developed insulin-loaded silk fibroin swelling microneedles (20 units per 0.5 sq.cm microneedle patch) for sustained drug release and delivery in vivo. The needles released insulin sustainably for 12 h and effectively controlled the blood glucose levels in diabetic rats. Thus, this microneedle system enables long-term hypoglycemic therapy [234]. Seong and coworkers developed double-layered microneedles with swelling needles enclosed in a non-swelling transdermal patch to improve skin adhesion. This interlocking mechanism resulted in a sustained release of insulin in vivo [232]. Courtenay et al. compared the efficiency of dissolving and swelling microneedles on the transdermal delivery of bevacizumab. Swelling microneedles provided a delayed and lower C_max_ than dissolving microneedles [233].

## 3. Clinical Trials of Microneedles for Biopharmaceutical Delivery

Numerous clinical trials have demonstrated the efficiency of microneedle systems in enhancing the transdermal delivery of various therapeutic agents, ranging from small molecules to macromolecules, vaccines, and particulate systems [54]. In a review, Dharadhar and coworkers discussed the applications and clinical trials of microneedles [59]. Statistically, a search on ClinicalTrials.gov for “microneedles” in October 2022 provided 138 results; among these, 88 studies have been completed. Clinical studies on human volunteers have been conducted for a variety of biomolecules, such as glucagon and insulin for diabetes, aflibercept and acetonide for diabetic macular edema, and parathyroid hormone for osteoporosis (Table 3) [157,207,235]. Several research groups have focused on transdermal delivery of insulin for diabetes (type 1 and 2) using pulled microneedles [169] and stainless steel microneedles [236,237], and reported that noninvasive microneedle application led to rapid drug delivery onset and excellent pharmacokinetic profiles [238]. Moreover, microneedle-mediated insulin delivery prevents the late hypoglycemic impact, provides consistent insulin levels in the blood, and minimizes inter-subject variation. A phase I clinical trial revealed that the 3M hollow microneedle system delivered a therapeutic dose of adalimumab (MW 148 kDa) more efficiently than the commercially available subcutaneous autoinjector (HUMIRA^®^) [239]. Clinical studies of the microneedle-assisted delivery of drugs for postmenopausal osteoporosis treatment (i.e., teriparatide and abaloparatide, and parathyroid hormone (PTH) 1-34) are also of significant interest to several companies, such as Corium, Zosano, Radius, and 3M.

## 4. Safety and Acceptability of Microneedles

### 4.1. Safety of Microneedles

In general, microneedle application only creates transient, reversible, superficial, and localized microinjuries (microchannels) in the skin, thus, several research groups have reported this noninvasive technique to have an excellent safety profile. The clinical safety of various microneedle types and safety aspects of microneedle materials have been reviewed elsewhere [31,59]. Microchannels do not allow the easy entrance of microorganisms into the skin tissue [240], thus leading to substantially less bacterial penetration than traditional hypodermic needles [241]. Furthermore, Quinn and colleagues revealed that microneedle-induced injuries in the skin healed and recovered quickly, minimizing the possibility of E. coli entering the skin [242]. Microneedle systems have been shown to be safe and effective in several clinical investigations [243]. To date, all short-term safety studies have revealed no epidermal or systemic infection incidence associated with microneedle insertion.

In actuality, even though many individuals have repeatedly treated their skin with cosmetic microneedle-based devices without device sterilization between uses, they reported no symptoms or experience of adverse effects (i.e., skin irritation or inflammation). Microneedles are generally considered safe for short-term usage, but repeated treatments may cause erythema and irritation, depending on the dimensions, geometries, and densities of the needles. Quinn et al. studied repeated insertion of dissolving microneedles and reported no safety issues owing to their safe and biocompatible material of construction [244]. In particular, the authors detected no acute inflammation or infection at the site of microneedle application. Further research is expected to study the safety of long-term or repeated usage of microneedles.

To ensure mechanical safety, microneedles should penetrate the skin without breakage during the insertion. The microneedles’ mechanical failure could cause biosafety issues (i.e., skin irritation and injuries) and inaccurate drug dosing. The mechanical safety of microneedles depends on the needles’ sharpness, aspect ratio, and material robustness [245,246]. The application of hollow microneedles could raise some technical issues of needle blockage and drug leakage. The dense skin tissue could partially or completely clog the needle bore, thereby hindering the fluid flow into the skin and reducing the drug delivery efficiency of hollow microneedles. High applied pressure or an insufficient needle penetration depth can result in fluid leakage on the skin surface, leading to drug wastage and inaccurate delivered doses.

Microneedle materials could be a critical factor for any potential toxic effects. Some safety concerns may arise when microneedle materials remain in the skin for an extended period, even though these selected materials are generally biocompatible and biodegradable. Unlike self-disabling dissolving and swelling microneedles, solid, hollow, and coated microneedles still generate some micron-sized sharp waste, thus creating a risk of cross-contamination and disease transmission [247]. However, such a risk is significantly lower for microneedles than conventional hypodermic needles. Additionally, individuals cannot reuse these microneedles due to the requirement of a specialized instrument to reload the medications onto the needles.

Several patients experience severe anxiety (needle phobia) while using traditional hypodermic needles to the extent that they even pass out. In a study on patients of all ages, 60% of children and 50% of adults reported being apprehensive of needles. Among these, pediatric subjects experienced significant aversion and stress [248]. The majority of pediatricians (84%) agreed that needle phobia is a serious clinical issue and that the use of hypodermic needles in children may impose a detrimental effect on their future interactions with healthcare providers [249]. Importantly, the microneedle technique eliminates the several potential safety risks and needle phobia of hypodermic needles, particularly those associated with child patients [250]. The capacity to mitigate injection anxiety is a major attraction of microneedles [251].

Research has shown dermal tolerance of microneedles with mild erythema as the most noticeable adverse effect [239]. Several pilot clinical investigations reported that noninvasive microneedle treatment caused no pain and slight irritation at most, while the skin completely recovered within a few hours. The risk of skin irritation increases significantly with the microneedle length, materials, and drug payload, among which the microneedle length is the most critical factor. The ability to cause no pain during the application is a favorable feature of microneedles. Researchers could quantitatively evaluate the pain level using a visual analog scale (VAS). Several factors could affect the pain level induced by microneedle insertion, such as needle dimensions, number, density, design, tip radius, and application site [67,252]. The VAS score was found to positively correlate with microneedle length, which had a markedly greater influence on pain than the needle number and density.

### 4.2. Acceptability of Microneedles

The widespread application and successful commercialization of microneedles depend on the acceptance of healthcare professionals and the general public’s interest and confidence in the products [251,253]. Evidently, the general public and care givers prefer microneedles to conventional hypodermic needles, demonstrating that this novel technology has been favorably embraced [254,255]. An ideal microneedle system should enable proper self-administration, requiring no or minimal training or involvement with trained medical professionals. Microneedle products have intrigued most children, who expressed a strong interest in the future application of microneedles, provided they gain confidence in the correct use of the device, safety, efficacy, and negligible discomfort. Clinical trials on human subjects reveal that naïve patients (without any former experiences with microneedle products) could insert microneedles into their skin successfully after receiving some basic instructions [256,257]. Patients have safely and effectively applied microneedles for transdermal drug administration at home with no incidence of side effects [235]. The primary driver of microneedle product adoption is the anticipated advantages of microneedles acknowledged by healthcare professionals and the general public [258]: painless application benefiting those with needle phobia, controlled drug delivery, self-administration, children’s preference, viable alternative to conventional routes of administration, low risk of needlestick injuries or bleeding, improved vaccination coverage, convenient disposal, and appealing product design.

## 5. Manufacturing and Regulatory Issues

### 5.1. Manufacturing Issues

Given the unique innovative design of microneedles, manufacturers would have to build specialized facilities for the mass production of microneedle products [257]. The high-precision manufacturing process would necessitate the use of micro-production techniques such as micromachining and nanoprocessing. The critical processes include precise machining, extrusion, and shaping of microneedles. During the early development stage, researchers usually fabricate microneedles manually in a modest number. These microneedles are sufficient for most phase I clinical and preclinical studies. When phase II trials are approaching quickly, thus requiring a large quantity of microneedle units, the manufacturing process should be more efficient and automated. When phase III clinical investigations conclude, the production will have achieved its maximum degree of automation, combined with expanded capacities and appropriate quality control systems [239].

The ideal design of microneedle products depends on various factors, including the availability of raw materials, the complexity of the device components, the number of production steps, and the viability of employing existing manufacturing methods and facilities. Typically, microneedle products must comply with the quality standards and regulations of both drug and medical devices. Quality assurance, quality control systems, and good manufacturing practice standards could impose a direct and significant impact on the production cycle. The manufacturers must address any issues related to mass production, including environment control (light, air, humidity, and temperature) and the management of chemistry, manufacturing, and controls (CMC). The selection and quality of materials are critical to the performance of finished microneedle products. The materials also play a significant role in determining whether the product will be approved or cleared by regulatory agencies. The use of safe, biocompatible, and thoroughly researched materials would save a great deal of time and cost, thus accelerating the development speed and efficiency. Furthermore, improvements in automation across the manufacturing processes are crucial for expanding production capacity and bolstering process control to ensure product quality. Additionally, a critical quality attribute of microneedles is the stability of the encapsulated therapeutic agents, especially thermosensitive macromolecules, which should be maintained during production, packaging, storage, transportation, and administration.

Microneedle sterilization has been a debatable topic among academic, industrial, and regulatory sectors; among these, industrial companies place more emphasis on this subject than academic institutions. There is no agreement on whether microneedle products have to be sterilized. The commonly used sterilization techniques (i.e., dry heat, steam, gamma or microwave radiation, ethylene oxide) could change the microneedle structure, compromise the needles’ mechanical strength, or degrade the loaded active ingredients. Some viable terminal sterilization strategies include ultraviolet light, gamma irradiation, ethylene oxide, or the addition of preservatives [259]. Notably, these methods should be compatible with the microneedle materials and protective towards the drug stability. Any companies in the pharmaceutical or medical device industries who are interested in the commercialization of microneedle products must invest substantial resources in the research, development, and optimization of the microneedle sterilization process. The producers must also carry out a risk assessment of bioburden control on microneedles. Several research groups have examined various sterilization techniques with the prospective requirements of regulatory authorities [260]. In general, it is critical to sterilize all raw materials, packaging materials, and production equipment to minimize contamination from workers or the environment. Furthermore, aseptic production will be time-consuming and challenging to execute if large-scale production is expected. Aseptic processing necessitates a clean environment and precise operating protocols, which contribute considerably to the cost and complexity of manufacturing processes. Researchers have reported several advantages of microneedle products during storage and transportation. Typically, a microneedle array, once assembled into a patch, may have a representative volume of roughly 1 cm^3^ [32,108]—substantially smaller than the size of a vial and needle–syringe package. Hence, microneedle patches could be easily and conveniently stored and distributed. Furthermore, microneedles do not require cold-chain storage, but rather these stable microneedle patches could be stored at room temperature, thus representing a significant cost saving.

### 5.2. Regulatory Issues

Microneedles provide some unique scientific and regulatory challenges, as these needles physically pierce the skin and compromise the skin barrier function. Microneedle products would face less opposition from regulatory agencies if they were viewed as a novel dosage form, instead of a subset of the currently available transdermal drug delivery systems [114]. There are currently no established, industry-wide regulatory requirements and standards for a ‘true’ microneedle product. Unfortunately, at this time, no ‘true’ microneedle product is commercially available on the market; therefore, no standards or guidelines ever exist for these products. This provides new complexities for mass production and highlights the necessity for widely recognized standards of quality control.

The US FDA has issued regulations specifying the conditions under which a microneedle product may be classified as a medical device, according to accessible information and its intended uses “in the diagnosis of disease or other conditions, or the cure, mitigation, treatment, or prevention of disease” or “to affect the structure or any function of the body of man”. In some cases, the regulatory agency may also classify microneedle products as drug delivery systems or consumer goods. The first microneedle product available on the market will provide a substantial source of information about the regulatory perspective. This will, in turn, set up the standards and requirements for successive microneedle products [261]. Standardized guidelines for production, characterization, evaluation, and quality control will considerably facilitate the commercialization of microneedles. The US FDA has organized several technical seminars in which researchers analyzed the standards and requirements of microneedle products [239,262]. In general, mass manufacturing of microneedles necessitates compliance with strict quality standards under the guidelines of the current good manufacturing practice and pharmaceutical quality system. Furthermore, the comprehensive evaluation should cover production parameters, in-process evaluation, document review, product specification, and examination of the finished product [261]. According to experts, the ICH quality guidelines provide practical recommendations for developing microneedle products’ chemical, manufacturing, and controls (CMC) data package [239]. The International Conference on Harmonization (ICH) Q6A guidance serves as the foundation for quality attributes of microneedle systems. Furthermore, ISO 11608 also specifies technical requirements to assess microneedle products [239]. Data packages supporting the CMC of microneedle products are necessary to meet the required criteria for pharmaceuticals and medical devices. Optimizing and validating the technology, and resolving regulatory issues, including long-term safety and sterility standards, are crucial for the development of microneedles in the future [263,264].

## 6. Conclusions

Historically, parenteral injection has been the most commonly used technique to administer biopharmaceutical products. However, this drug delivery method has some significant drawbacks (i.e., needle phobia, sharp waste, disease transmission, etc.). As a viable alternative, transdermal delivery resolves most technical issues associated with hypodermic needles, thus improving patient compliance and acceptability. An efficient skin permeation generally requires some special properties of the permeants (i.e., low molecule weight, high potency, and moderate lipophilicity). With the development of physical enhancement technologies, the transdermal delivery of ‘difficult’ macromolecules becomes feasible. Interestingly, microneedle systems exhibit excellent potential as a platform to deliver biotherapeutics into and across the skin. With the aid of microneedles, biomolecules could be more stable and penetrate the skin at the therapeutic dose, thus improving the therapy efficacy. Several academic institutions and industrial companies have developed and evaluated microneedle-mediated delivery systems for various biomolecules. While most researchers have employed hollow microneedles to administer biopharmaceutical drugs, some recent investigations have been focusing on the application of polymeric dissolving microneedles. The published findings on microneedles indicate that these systems may be adapted into self-administered devices, thus equipping patients with more convenience and independence.

## 7. Future of Microneedles

Further improvements to current microneedle-based devices will make the transdermal delivery of biomolecules feasible and achievable. Several factors would determine the feasibility of a microneedle product to enter the commercial market, including drug stability, long-term safety, dose restriction, efficient drug delivery, GMP compliance, manufacturability, and scaling-up process. Furthermore, effective marketing strategies would allow microneedle products to capture a significant market share. In the future, scientists will further investigate drug metabolism in the skin, the breakdown of microneedle materials, and long-term adverse effects and safety issues. Moreover, researchers will endeavor to develop innovative and effective designs and build simulation modelling for microneedle penetration and drug diffusion. For the successful clinical translation of microneedle products, we envisaged the creation of advanced materials (for targeted drug delivery and simple manufacturing processes) and optimization of efficient industrial-scale production techniques (to lower production cost, simplify processes, and save time). A large number of research publications and preclinical and clinical trials are advancing microneedle production towards the large commercial scale. Notably, the rapid advancement in high-resolution 3D printing technology could facilitate a low-cost, simple, robust, reproducible, customizable, and scalable mass production of microneedles.

Large-scale microneedle manufacturers will invest substantial resources in optimizing production processes, minimizing technical errors, simplifying the sterilization process, and increasing the drug-loading capacity of microneedles. Recently, the research, development, market, and clinical applications of microneedles have been growing exponentially. Therefore, the accelerated development of microneedle products is expected to have a positive impact on patients’ lives, public health, and economic aspects. The technological advancement will result in the creation of innovative delivery systems that have low costs, small dimensions, low required doses, lesser side effects, and high acceptability to deliver various biopharmaceuticals transdermally [265]. In actuality, several pharmaceutical companies are postponing their microneedle development until the success of the first-to-market microneedle drug delivery product becomes evident, due to the costly manufacturing and uncovered issues (i.e., technical and regulatory issues) of microneedles. Regulatory requirements (i.e., sterility, packaging, disposal, administration, and long-term stability) constitute a significant obstacle to the commercial manufacturing of microneedles. The eventual success of microneedle products depends heavily on their functionality, and acceptability from healthcare providers and the public at large. Furthermore, we expect a strong collaboration between academia, industry, inventors, patients, and regulators for the development of microneedle products, with commercial viability functions as the driving force for the growth of this field. As the number of approved macromolecules increases rapidly, the application of microneedle-based delivery systems will expand tremendously, thus gradually substituting traditional dosage forms and administration techniques [266]. These efforts will lead to the arrival of several commercial and marketable microneedle products, thus unleashing the bright and prominent future of microneedle technology. Consequently, the use of microneedle systems will lead to a paradigm shift in the field of drug delivery, especially for those therapeutic agents that have previously been inaccessible with traditional techniques. As anticipated, microneedle technology will enable personalized medicines that improve patients’ quality of life and medications’ therapeutic effects.

## Figures and Tables

**Table 1:** Biomolecule degradation in microneedles [32].

Factor Types	Factors	Degradation	Microneedle Types
Drug–device interface	Needle bore, material properties, surface morphology, drug formulation	Aggregation, adsorption, unfolding	Hollow, coated, and solid microneedles
Drug concentration	Coating and molding formulation	Aggregation	Coated, dissolving, and swelling microneedles
Elevated temperature	Material polymerization, transition temperature, drying process	Aggregation, chemical degradation, unfolding	Coated and dissolving microneedles
Metal catalysis	Formulation ingredients, microneedle materials	Aggregation, oxidation	Hollow and solid metal microneedles
Air exposure	Storage conditions	Aggregation, adsorption, oxidation, unfolding	Coated, dissolving, swelling, and solid microneedles
pH	Composition and properties of coating and molding formulation	Aggregation, chemical degradation, unfolding	Coated, dissolving, swelling, and solid microneedles

**Table 2:** Microneedle types for transdermal drug delivery.

Microneedle Types	Microneedle Processes for Drug Delivery	Advantages	Disadvantages
Solid microneedles	“*Poke and patch*” *technique* Microneedle fabrication Preparation of drug formulation Characterization of microneedles Characterization of drug formulation Skin insertion of microneedles Removal of solid microneedles Characterization of microchannels Application of drug formulation on microneedle-treated area Drug permeation study	Mechanically robust microneedlesMicroneedles could be fabricated in harsh conditions and from various materials Simple production Versatile drug formulations Delivery of large doses Use for molecules with high and low potency Possibility of sustained drug delivery	Significant effects of pore closure Complicated two-step application Possible error of misalignment of microneedle treatment and formulation application sites Biosafety risk of microneedle fracture in skin tissue Sharp waste disposal Risk of disease transmission Risk of microneedle reuse Significant drug loss/waste (low fraction of drug delivered) No accurate dosing Thermolabile drugs in liquid or semisolid formulations require cold-chain storage and transportation Slow drug release by diffusion Long wearing time Separate packages for microneedles and formulation
Hollow microneedles	“*Poke and flow*” *technique* Microneedle fabrication Preparation of drug formulation Characterization of microneedles Characterization of drug formulation Skin insertion of microneedles Injection of drug formulation Characterization of microchannels Drug permeation study	Simple one-step application Microneedles could be fabricated in harsh conditions Convenient production by downscaling hypodermic needlesDelivery of large and accurate doses Controlled rate of drug delivery Use for molecules with high and low potency No requirement for drug reformulation High delivery efficiency	Fabricated from only strong materials to ensure the microneedle robustness Limited microneedle designs Biosafety risk of fracture of weak microneedles in skin tissue Possible bore clogging Possible drug leakage Extended wearing time Risk of microneedle reuse Risk of disease transmission Complex two-component device: microneedles and drug reservoir Drug formulation limited to low-viscosity solution Sharp waste disposal Thermolabile drugs in liquid formulation require cold-chain storage and transportation
Coated microneedles	“*Coat and poke*” *technique* Fabrication of solid microneedles Preparation of coating formulation Characterization of solid microneedles Characterization of coating formulation Coating drug formulation onto microneedles’ surface Dissolution of coated layer and drug release kinetics Skin insertion of microneedles Characterization of microchannels Drug permeation study	Simple one-step application Improved drug stability in solid form Versatile polymers for coating formulation No requirement for cold-chain storage and transportation Mechanically robust microneedlesNo risk of microneedle reuse Rapid dissolution, fast drug release Short wearing time Precise dosing High delivery efficiency Single product package	Controlled, mild production environment Limited drug-coating quantity and delivery dose Suitable for highly potent molecules Sharp waste disposal Risk of disease transmission Risk of drug dislocation on microneedle array Coating layer affects needle sharpness and skin penetration efficiency Requirement for drug reformulation
Dissolving microneedles	“*Poke and release*” *technique* Preparation of drug-loaded polymeric formulation Characterization of polymeric formulation Fabrication of drug-loaded microneedles Characterization of dissolving microneedles Dissolution of microneedles and drug release kinetics Skin insertion of microneedles Characterization of microchannels Drug permeation study	Simple one-step application Improved drug stability in solid form No requirement for cold-chain storage and transportation No risk of microneedle reuse No risk of disease transmission No sharp waste Short wearing time Microneedle dissolution depends on formulation and materials Possibility of bolus or sustained drug release and delivery Minimal drug loss during fabrication and application Precise dosing High delivery efficiency Single product package	Limited range of materials with sufficient mechanical strength, biocompatibility, and biodegradability Limited drug-loading quantity and delivery dose Suitable for highly potent molecules Drug payload affects microneedles’ mechanical strength and sharpness Requirement for drug reformulation
Swelling microneedles	“*Poke and swell*” *technique* Fabrication of swelling microneedles Preparation of drug formulation Characterization of swelling microneedles Characterization of drug formulation Drug release kinetics from the reservoir Skin insertion of drug-reservoir-assembled swelling microneedles Characterization of microchannels Drug permeation study	Simple one-step application Improved drug stability in solid form No requirement for cold-chain storage and transportation No risk of microneedle reuse No risk of disease transmission No sharp waste No biosafety risk Delivery of large doses Microneedles’ mechanical strength and sharpness unaffected by the drug payload Use for molecules with high and low potency Single product package	Limited range of swelling materials Requirement for drug reformulation Low delivery efficiency, low fraction of drug delivered No accurate dosing Slow drug release by diffusion Long wearing time

**Table 3:** Clinical trials of microneedle-mediated delivery of macromolecules.

NCT No.	Clinical Trial	Condition and Diseases	Drug and Device	Phase	Location	Status
NCT00837512	Insulin delivery using microneedles in type 1 diabetes	Type 1 diabetes mellitus	Device: hollow microneedle (1 mm) Device: subcutaneous (SC) insulin catheter	II, III	Emory University (USA)	Completed
NCT02837094	Enhanced Epidermal Antigen-Specific Immunotherapy Trial-1 (EE-ASI-1)	Type 1 diabetes	Drug: C19-A3 GNP (MicronJet 600)	I	Cardiff University	Unknown
NCT02329457	VZV Vaccine for Hematopoietic Stem Cell Transplantation (VZIDST)	Varicella Zoster infection	Biological: Zostavax	II, III	The University of Hong Kong	Completed
NCT03274674	Use of Injectable-Platelet-Rich-Fibrin (I-PRF) to Thicken Gingival Phenotype	Periodontoclasiagingiva; injury condition blood clot gingiva disorder	Other: I-PRF	NA	Bezmialem Vakif University (Turkey)	Completed
NCT00602914	A pilot study to assess the safety, PK, and PD of insulin injected via MicronJet or conventional needles	Diabetes mellitus	Device: MicronJet Device: conventional needle (NanoPass microneedle)	Early Phase I	NanoPass Technologies Ltd.	Completed
NCT02459938	Safety and Efficacy of ZP-Glucagon to Injectable Glucagon for Hypoglycemia	Hypoglycemia	Solid/metal (drug-coated titanium microneedles) Zosano microneedle patch	I	Nucleus Network (Australia)	Completed
NCT00489918	Dose-ranging study—Macroflux parathyroid hormone (PTH) in postmenopausal women with osteoporosis	Osteoporosis	Drug: Teriparatide (Zosano Pharma)	II	Zosano Pharma Corporation	Completed
NCT02478879	A study to determine the patient preference between Zosano Pharma parathyroid hormone (ZP-PTH) patch and the Forteo pen	Postmenopausal osteoporosis	Coated titanium (ZP-PTH microneedle patch)	I	Covance Daytona Beach Clinical Research Unit (USA)	Completed
NCT01674621	Phase 2 study of BA058 (Abaloparatide) transdermal delivery in postmenopausal women with osteoporosis	Postmenopausal osteoporosis	Drug: BA058 placebo Drug: BA058 TD (50, 100, 150 µg) Drug: BA058 injection (80 µg) (TD: coated 3M microstructured transdermal system (MTS), 250 µm, 316 microprojections)	II	Radius Health, Inc.	Completed
NCT03607903	Adalimumab microneedles in healthy volunteers	Pain injection site	Biological: Adalimumab ID or SC Biological: Adalimumab SC Other: saline ID or SC (3M hMTS, 1500 µm, 12 needles)	I, II	Centre for Human Drug Research (Netherlands)	Completed
NCT03054480	Fractional Micro-Needle Radiofrequency and I Botulinum Toxin A for Primary Axillary Hyperhidrosis	Primary axillary hyperhidrosis	Device: fractional microneedle radiofrequency Drug: botulinum toxin type A	NA	Thep Chalermchai, Mae Fah Luang, University Hospital	Completed
NCT03126786	Suprachoroidal CLS-TA With Intravitreal Aflibercept Versus Aflibercept Alone in Subject with Diabetic Macular Edema	Diabetic macular edema	IVT aflibercept, Sham SC, SC CLS-TA	II	Clearside Biomedical, Inc.	Completed
NCT03203174	The use of microneedles with topical botulinum toxin for the treatment of palmar hyperhidrosis	Hyperhidrosis	Solid (Sham microneedle) Botulinum toxin type A	I	University of California, Davis	Completed
NCT01684956	Pharmacokinetic comparison of intradermal versus subcutaneous insulin and glucagon delivery in type 1 diabetes	Type 1 diabetes	Hollow (MicronJet™)	II	Massachusetts General Hospital (USA)	Unknown
NCT01557907	Multi-day (three) in-patient evaluation of intradermal versus subcutaneous basal and bolus insulin infusion	Diabetes	Hollow (BD research catheter)	I/II	Profil Institut fur Stoffwechselfforschung GmbH (Germany)	Completed
NCT01120444	Study on the effects on blood glucose following intradermal and subcutaneous dosing of insulin in diabetic patients	Diabetes	Hollow (BD research catheter)	I/II	Profil Institute of Clinical Research (Germany)	Completed
NCT01061216	Pharmacokinetics/dynamics of basal (continuous) insulin infusion administered either intradermally or subcutaneously	Diabetes Mellitus, Type 1/2	Hollow (BD research catheter)	I/II	Profil Institut fur Stoffwechselfforschung GmbH (Germany)	Completed
NCT00553488	Feasibility Study of the Effect of Intra-Dermal Insulin Injection on Blood Glucose Levels After Eating	Diabetes mellitus, type 1	BD research catheter (34G × 1.5 mm needle) Insulin	II	Profil Institut fur Stoffwechselfforschung GmbH (Germany)	Completed
NCT01518478	Atopic Dermatitis Research Network (ADRN) Influenza Vaccine Pilot	Atopic dermatitis	Fluzone^®^ intradermal	I	National Institute of Allergy and Infectious Diseases (USA)	Completed
NCT01737710	Atopic Dermatitis Research Network (ADRN) Influenza Vaccine Study	Atopic dermatitis	Fluzone^®^ intradermal vaccine Fluzone^®^ (intramuscular) vaccine	I	National Institute of Allergy and Infectious Diseases (USA)	Completed
NCT04064411	Efficacy and Safety of Abaloparatide-Solid Microstructured Transdermal System in Postmenopausal Women With Osteoporosis	Postmenopausal osteoporosis	Abaloparatide solid microstructured transdermal system; abaloparatide-SC	III	Radius Health, Inc.	Completed

NA: Not Applicable.

## Abbreviations

bFGF	Basic fibroblast growth factor
BSA	Bovine serum albumin
CDC	Centers for Disease Control and Prevention
CMC	Chemistry, manufacturing, and controls
FDA	Food and Drug Administration
FITC	Fluorescein isothiocyanate
hMTS	Hollow microstructured transdermal system
ICH	International Conference on Harmonization
IgG	Immunoglobulin G
MN	Microneedle
MW	Molecular weight
PLA	Polylactic acid
PLGA	Poly Lactic-co-Glycolic Acid
PTH	Parathyroid hormone
PVA	Polyvinyl alcohol
rhGH	Recombinant human growth hormone
sMTS	Solid microstructured transdermal system
UV	Ultraviolet
VAS	Visual analog scale
